# Holotoxin disassembly by protein disulfide isomerase is less efficient for *Escherichia coli* heat-labile enterotoxin than cholera toxin

**DOI:** 10.1038/s41598-021-03939-9

**Published:** 2022-01-07

**Authors:** Albert Serrano, Jessica L. Guyette, Joel B. Heim, Michael Taylor, Patrick Cherubin, Ute Krengel, Ken Teter, Suren A. Tatulian

**Affiliations:** 1 Burnett School of Biomedical Sciences, College of Medicine, University of Central Florida, Orlando, FL 32816 USA; 2 Department of Chemistry, University of Oslo, Blindern, Oslo, Norway; 3 Department of Physics, College of Sciences, University of Central Florida, Orlando, FL 32816 USA; 4 Present Address: Viral Pathogenesis Section, Laboratory of Viral Diseases, National Institutes of Health, Bethesda, MD USA; 5 Present Address: Department of Microbiology, Perelman School of Medicine, University of Pennsylvania, Philadelphia, PA USA

**Keywords:** Biochemistry, Biophysics, Molecular biology

## Abstract

Cholera toxin (CT) and *Escherichia coli* heat-labile enterotoxin (LT) are structurally similar AB_5_-type protein toxins. They move from the cell surface to the endoplasmic reticulum where the A1 catalytic subunit is separated from its holotoxin by protein disulfide isomerase (PDI), thus allowing the dissociated A1 subunit to enter the cytosol for a toxic effect. Despite similar mechanisms of toxicity, CT is more potent than LT. The difference has been attributed to a more stable domain assembly for CT as compared to LT, but this explanation has not been directly tested and is arguable as toxin disassembly is an indispensable step in the cellular action of these toxins. We show here that PDI disassembles CT more efficiently than LT, which provides a possible explanation for the greater potency of the former toxin. Furthermore, direct examination of CT and LT domain assemblies found no difference in toxin stability. Using novel analytic geometry approaches, we provide a detailed characterization of the positioning of the A subunit with respect to the B pentamer and demonstrate significant differences in the interdomain architecture of CT and LT. Protein docking analysis further suggests that these global structural differences result in distinct modes of PDI-toxin interactions. Our results highlight previously overlooked structural differences between CT and LT that provide a new model for the PDI-assisted disassembly and differential potency of these toxins.

## Introduction

**Figure 1:**
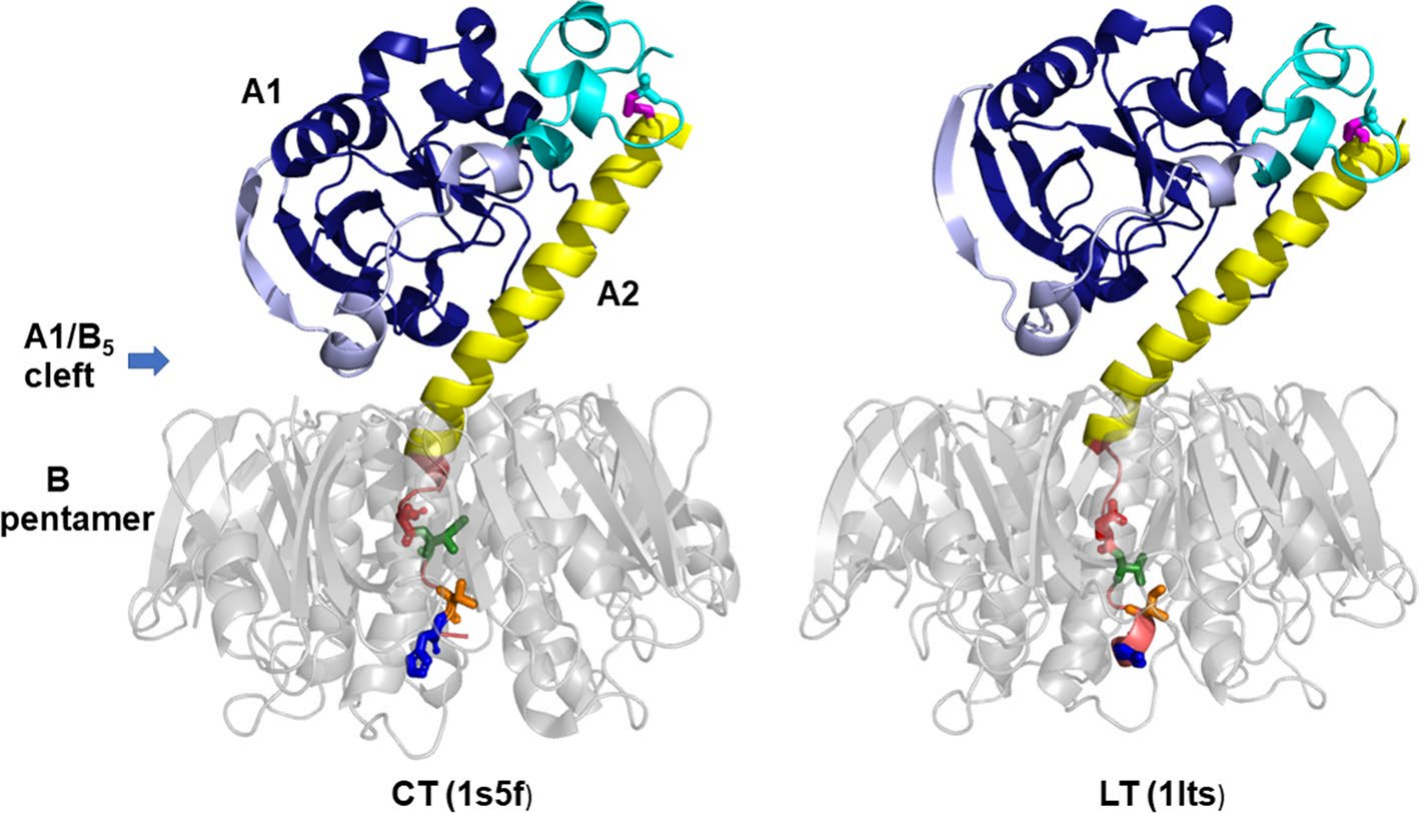
CT and LT structures. CT and LT are composed of a catalytic A1 subunit, a linker A2 subunit, and a cell-binding B pentamer (grey) that is organized as a ring-like structure from five identical subunits. The A1 subunit has three distinct subdomains: A1-1 (dark blue) spans residues 1–132 and represents the catalytic core of the protein, while A1-2 (light blue) spans residues 133–161 and wraps around the A1-1 subdomain to form an extended bridge with the A1-3 subdomain (cyan), a globular region that spans residues 162–192. A disulfide bond (magenta) connects cysteine 187 in the A1-3 subdomain with cysteine 199 in the N-terminal region of the A2 subunit. The major α-helical component of the A2 subunit (yellow) extends into the opening of the B pentamer, where it bends and threads through the pore in a largely unstructured state. The differences in CT and LT toxicity have been mapped to an 11 amino acid region of the non-catalytic A2 subunit that is located within the pore of the B pentamer (mauve). The four amino acid differences between CTA2 and LTA2 in this region are presented in stick format, with residues 229, 230, 232, and 233 colored red, green, orange, and blue, respectively. These residues are Asp, Ile, Thr, His in CT and Glu, Val, Ile, Tyr in LT. Images are generated by PyMOL 1.8.6.0 (Schrödinger, LLC).

Cholera toxin (CT) and *Escherichia coli* heat-labile enterotoxin (LT) are AB_5_-type protein toxins that share ~ 82% amino acid sequence identity and a common toxicity mechanism^1,2^. They are composed of a receptor-binding pentamer of B subunits (B_5_) and an A subunit (Fig. 1). The A subunit is produced as one chain and comprises an enzymatic A1 portion (amino acid residues 1–192 for CT or 1–194 for LT) and an A2 portion (residues 193–240 for CT or 195–240 for LT) that enters the central pore of the B_5_ pentamer by its C-terminal tail and thereby provides a noncovalent linkage between the A and B_5_ portions. The A chain is proteolytically nicked into A1 and A2 parts, which stay connected through a disulfide bond between Cys187 and Cys199. Reduction of the A1-A2 disulfide bond in the host cell is required for toxin activation and release of the A1 chain into the cytosol, where it catalyzes the ADP-ribosylation of Gsα. This results in cAMP-mediated electrolyte and fluid leakage in the intestine, severe dehydration, and diarrhea^3^.

The atomic-resolution structures of various forms of CT and LT, such as wild type or mutant proteins, with or without bound ligands, different crystal forms, have been determined by X-ray crystallography^4–11^. Both toxins adopt overall very similar structures, with a B_5_ ring and an A subunit tethered on top like a wedge. The A2 chain runs as a continuous α-helix down to the orifice of the B_5_ central opening and threads through the pore. Despite these common conformational features, significant structural differences have been reported for the C-terminal tails of the A2 subunits of LT and CT. In the LT structure, the A2 tail spans the B_5_ pore as an extended chain, with a 1.5-turn helix at the C-terminus^4–9^ (Protein Data Bank (PDB) IDs 1lt4, 1lta, 1ltg, 1lti, 1lts, and 1ltt). In contrast, the C-terminal segment of A2 in the first structure of CT (PDB ID 1xtc) assumes α-helix-like structure that makes extensive, mostly nonpolar contacts with the pore, thus stabilizing the whole structure^11^. However, this structure has poor geometry and is not well refined. Later X-ray crystallography studies of CT show a divergent conformation for the pore-spanning A2 tail which involves an elongated, non-helical structure with a 1-turn helix at the end^10^ (PDB IDs 1s5b, 1s5c, 1s5d, 1s5e, and 1s5f). The last four or five amino acids of the A2 subunit are missing in these structures because of poor electron density.

The functional implications of these structural details have been explored in several studies. Cell culture experiments on polarized T84 intestinal epithelial cells showed that CT causes Cl^-^ ion secretion with nearly twofold faster kinetics as compared to LT^12,13^. Extensive studies of mutant and chimeric proteins concluded that the region responsible for the difference in toxicity resides in an 11 amino acid sequence (226–236) near the C-terminus of the A2 subunit, where 4 residues differ between CT and LT (D229, I230, T232, and H233 for CTA2; E229, V230, I232, and Y233 for LTA2, see Fig. 1)^13^. It was proposed that the A2 tail provides CT with a greater stability than LT, which consequently allows more CTA1 to be delivered into the host cytosol. The relative stabilities of CT and LT were not directly tested, however. The supporting evidence for this hypothesis was derived from studies on CT/LT hybrid toxins rather than the two native toxins, with the hybrid toxin serving as a proxy for CT exhibiting greater in vitro stability and more potent cellular activity than the hybrid toxin representing LT^13^. Still, a molecular dynamics analysis suggested more efficient nonpolar contacts between the A2 tail and the central pore of the B pentamer and restricted solvent intercalation into the pore for CT as compared to LT, possibly contributing to the predicted higher cellular stability of CT^14^. This conjecture is disputable, given the fact that holotoxin disassembly is a critical step for toxicity.

Holotoxin disassembly occurs after CT and LT travel from the cell surface to the endoplasmic reticulum (ER) of a target cell. Reduction of the A1/A2 disulfide bond by glutathione occurs at the resident redox state of the ER^15^, but reduction alone does not release the A1 subunit from the rest of the toxin^16^: disassembly specifically requires the action of protein disulfide isomerase (PDI)^17,18^. Cells lacking PDI from RNAi are thus completely resistant to both CT^18^ and LT (manuscript in preparation). PDI-driven toxin disassembly is hypothesized to occur by a physical mechanism in which the A1 subunit is pushed away from the A2/B_5_ complex by the expanded structure of PDI that results from its contact with the A1 subunit^19^. The dissociated A1 subunit can then enter the cytosol where it modifies its Gsα target.

In this work, we adopt a new approach to the differential toxicity of CT and LT and its relation to the structural features of the toxins. We show that PDI disassembles CT more efficiently than LT, and this correlates with the greater cellular potency of CT in comparison to LT. CT and LT did not exhibit differences in stability when subjected to an ELISA that had previously detected differences in the stabilities of two CT/LT hybrid toxins used as proxies for native CT and native LT. Quantitative description of the A subunit orientation for six CT and six LT structures, using novel analytic geometry simulations, unveils a significant difference between the tilt angles of the A2 α-helix with respect to the B pentamer plane for the two toxins. This gross angular difference results in distinct positioning of the A subunit for CT and LT, an important and previously overlooked structural feature. Lastly, protein docking analysis suggests that these tertiary structural differences between LT and CT constitute a key factor modulating the mode of PDI-toxin interaction, toxin disassembly and toxicity.

## Results

### CT and LT differ in toxicity and PDI-driven toxin disassembly but not overall stability

**Figure 2:**
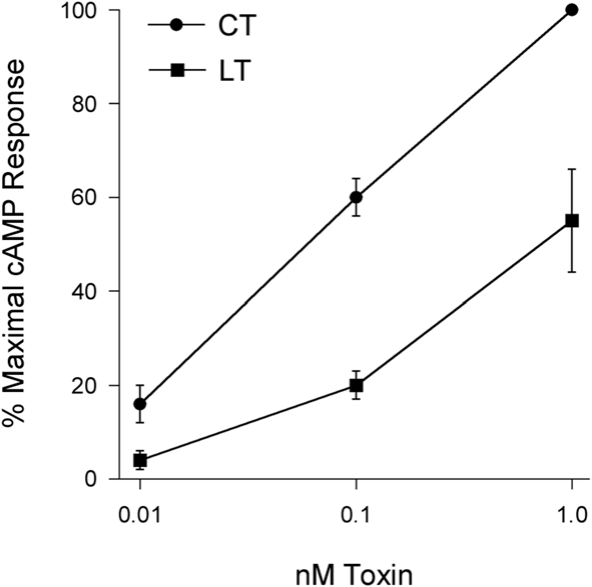
Comparative analysis of CT/LT potency. CHO cells were incubated with 0.01 nM, 0.1 nM, or 1.0 nM CT or LT for 2 h before cAMP levels were quantified. Data are expressed as percentages of the maximal cAMP response for the assay and are presented as mean ± standard error of six independent experiments with triplicate samples.

We first verified the differential cellular activities of the toxin preparations. LT from a human enterotoxigenic *Escherichia coli* strain and CT were both purified as recombinant proteins using *E. coli* C43(DE3) cells. CT exhibited greater potency than LT in producing cAMP in cultured CHO cells (Fig. 2). At the highest toxin concentration, the LT-induced cAMP response was ~ 50% of that induced by CT and was roughly equivalent to the response obtained with a tenfold lower concentration of CT. Thus, both toxins were active and displayed the expected distinct cellular potencies.

**Figure 3:**
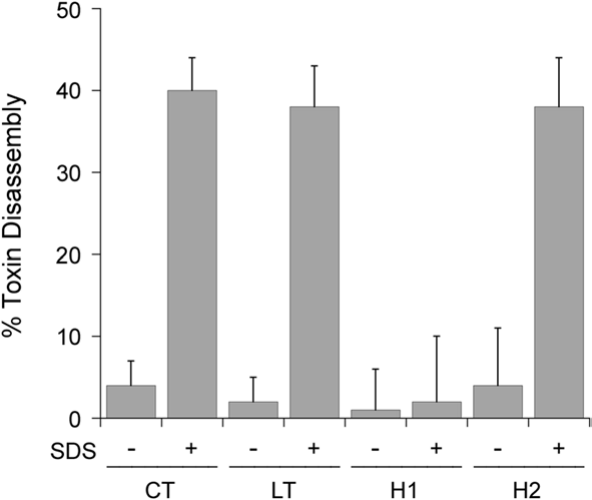
Comparative analysis of CT/LT stability. The indicated toxins were appended to the wells of a GM1-coated ELISA plate and exposed to pH 5.5 medium in the absence or presence of 0.1% SDS for 30 min at 25 °C. After extensive washing, sequential incubations with primary and HRP-conjugated secondary antibodies were used to detect the A1 subunit. The percentage of toxin disassembly was calculated from the maximum A1 signal obtained from the corresponding untreated holotoxin. H1 is a hybrid toxin that was previously thought to mimic native CT^13^; H2 is a hybrid toxin that was previously thought to mimic native LT^13^. Error bars present the standard error of the means from five independent experiments with six replicate wells per condition.

Different potencies for CT and LT were originally attributed to the more stable A2/B_5_ contact for CT than for LT. However, the data supporting this model were generated with CT/LT hybrid toxins rather than the native toxins^13^. We therefore compared the relative stabilities of CT and LT using the same ELISA that was previously applied to the CT/LT hybrid toxins^13^ (Fig. 3). For this assay, we also generated our own versions of the two CT/LT hybrid toxins that were previously studied: the H1 hybrid contained the CTA subunit and the LTB pentamer, while the H2 hybrid contained the CTA1 subunit, a mutant CTA2 subunit with the LTA2 sequence between residues 226–236, and the LTB pentamer. Our hybrids contained a C-terminal KDEL sequence as opposed to the RDEL sequence in the original hybrid toxins, but this difference does not contribute to differential toxicity^13^. CT, LT, and the two hybrid toxins were each appended to a 96-well plate coated with GM1 ganglioside, which is the cell surface receptor for both toxins. The high-affinity interaction between GM1 and the B pentamer prevents toxin release from the plate and places the holotoxin in a biologically meaningful orientation. An antibody recognizing the A1 subunit was then used, along with a secondary antibody conjugated to horseradish peroxidase (HRP) and 3,3′,5,5′-tetramethylbenzidine (TMB) substrate, to document the presence of the toxin-anchored A1 subunit. The ELISA was also performed with parallel samples of toxin that had first been exposed to pH 5.5 medium or 0.1% SDS in pH 5.5 medium. Acidified medium alone did not result in an appreciable loss of A1 subunit from any toxin, which was consistent with the previous study that only used the H1 and H2 toxins^13^. That study reported the addition of 0.5% SDS to pH 5.5 medium was required to detect a difference in toxin stability, with the H1 hybrid exhibiting a greater level of stability (i.e., less disassembly) than the H2 hybrid. We replicated that general observation here, recording essentially no disassembly of the H1 hybrid and ~ 40% disassembly of the H2 hybrid in pH 5.5 medium containing 0.1% SDS. However, we found no difference between the disassembly of CT and LT under the same condition. Both wild-type toxins exhibited the same level of stability, which was comparable to the stability of the H2 hybrid. These data question the validity of extrapolating the stabilities of H1 (thought to represent CT^13^) and H2 (thought to represent LT^13^) to the stabilities of native toxins. Factors other than toxin stabilities must therefore be responsible for the distinct cellular potencies of CT and LT.

**Figure 4:**
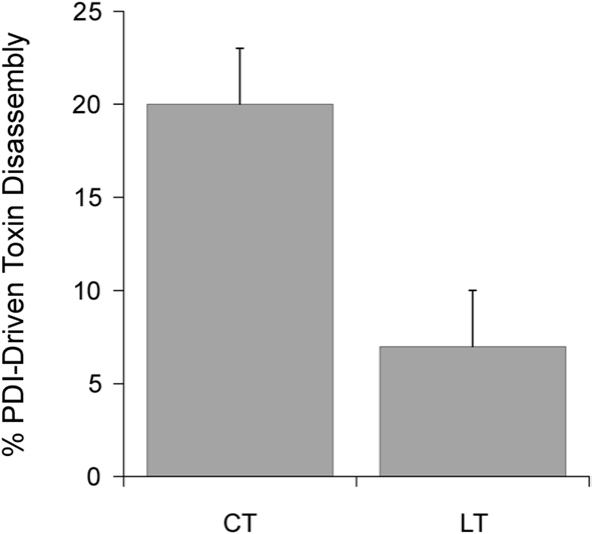
Comparative analysis of CT/LT disassembly by PDI. CT and LT were appended to the wells of a GM1-coated ELISA plate and exposed to reduced PDI for 1 h at 37 °C. After extensive washing, sequential incubations with primary and HRP-conjugated secondary antibodies were used to detect the A1 subunit. The percentage of toxin disassembly was calculated from the maximum A1 signal obtained from the corresponding untreated holotoxin. Error bars present the standard error of the means from four independent experiments with six replicate wells per condition.

PDI is responsible for the disassembly of CT and is necessary for CT intoxication^18^. PDI is also required for LT intoxication (manuscript in preparation), but the PDI-driven disassembly of LT has not been documented. We accordingly examined the efficiency of CT and LT disassembly by PDI with an ELISA protocol similar to the toxin stability assay (Fig. 4). The toxins were again captured in a 96-well plate coated with GM1. For this assay, however, the toxins were exposed to PDI rather than acidified medium with detergent. An interaction with PDI releases the A1 subunit from the plate-anchored A2/B_5_ complex, which is detected through the loss of A1 signal^20^. With this assay, we found that PDI disassembles CT with greater efficiency than LT. A 1 h incubation with PDI released 20 ± 3% of CTA1 from its holotoxin, whereas only 7 ± 3% of LTA1 was displaced from its holotoxin. This was a statistically significant difference, with a *p* value of 0.015 (Student's t test). The higher cellular potency of CT thus correlates with more efficient PDI-driven toxin disassembly for CT than for LT.

**Figure 5:**
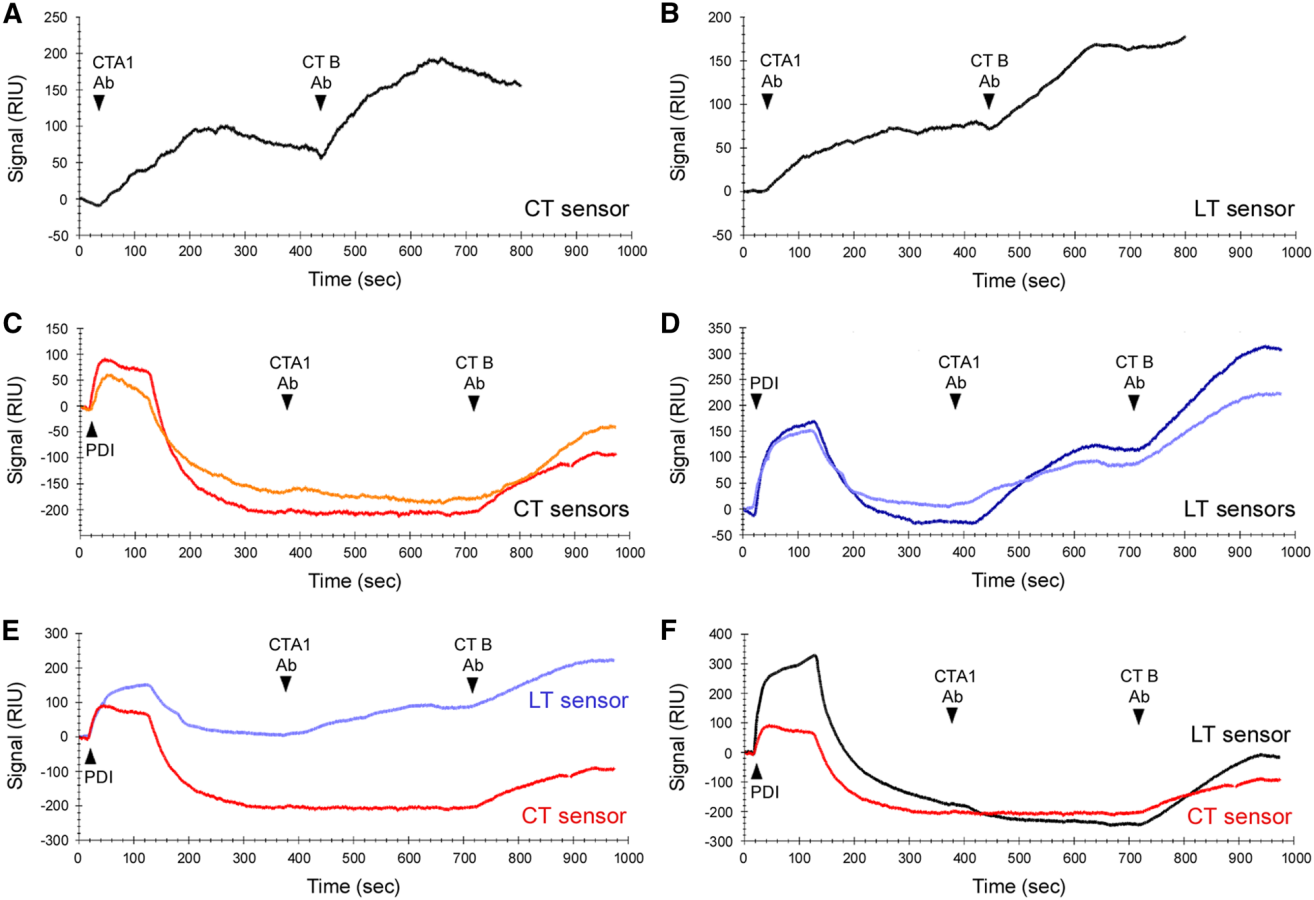
Relative kinetics of CT/LT disassembly by PDI. CT or LT was appended to a GM1-coated SPR sensor, and a baseline measurement corresponding to the mass of the sensor-bound holotoxin was taken to establish the 0 RIU signal. (**A-B**) Antibodies against the CTA1 and CTB subunits were perfused over a CT-coated (**A**) or LT-coated (**B**) sensor as indicated. (**C,D**) Buffer containing 1 μM PDI and 1 mM DTT was added to a CT-coated (**C**) or LT-coated (**D**) sensor. This was followed by sequential injections of antibodies against the A1 and B subunits, as indicated. Results from two independent experiments are shown for each toxin. (**E**) Traces for one CT disassembly experiment (red, from **C**) and one LT disassembly experiment (light blue, from **D**) are overlaid for comparative purposes. (**F**) An LT disassembly experiment performed with 10 μM PDI in the perfusion buffer (black) is overlaid with a CT disassembly trace (red, from **C**).

A second assay based on surface plasmon resonance (SPR) measurements confirmed the differential disassembly of CT and LT by PDI (Fig. 5). The toxins were captured on GM1-coated SPR sensors, with preliminary measurements establishing the baseline refractive index unit (RIU) corresponding to the mass of the bound holotoxin. This baseline was set as 0 RIU. Control experiments confirmed antibodies against CTA1 and CTB recognized both CT (Fig. 5A) and LT (Fig. 5B), which was expected given the high level of identity between the two toxins^1^.

Binding was documented through the elevated RIU signals generated after antibody perfusion over the toxin-captured sensor. If toxin disassembly occurred upon exposure to PDI, the A1 subunit would be released from its holotoxin and removed from the sensor by the flow of the perfusion buffer. The RIU would consequently drop to a point below the starting value of the intact holotoxin, and no signal would be generated by subsequent injection of the CTA1 antibody. We have previously reported these observations for PDI-treated CT^18–22^ and replicated the result here: after an increase in RIU resulting from the binding of PDI to CT, the signal dropped significantly below the initial baseline value (Fig. 5C). Exposure of the PDI-treated toxin to sequential injections of CTA1 and CTB antibodies only produced a signal for the CTB antibody. This demonstrated that the A1 subunit had been specifically, and completely, removed from the sensor. Toxin disassembly was dependent upon the presence of PDI, as an intact holotoxin remained on the sensor, with no loss of the baseline RIU signal, when CT was exposed to 1 mM DTT alone (see Supplementary Fig. S1). For LT, the elevated RIU signal initially resulting from PDI binding only dropped to around the starting baseline level (Fig. 5D). Elevated signals were generated from subsequent injections of the A1 and B antibodies, indicating the presence of a large fraction of intact holotoxin on the LT sensor 400 s after its exposure to PDI. A direct comparison of the LT and CT data highlighted the lower efficiency of LT disassembly in comparison to CT disassembly (Fig. 5E). The use of 10 μM PDI improved the kinetics and extent of LT disassembly, such that it closely approximated the kinetics of CT disassembly by 1 μM PDI and resulted in a complete loss of LTA1 from the sensor (Fig. 5F). These data documented the PDI-driven disassembly of LT, but the complete disassembly of LT required a higher concentration of PDI than was necessary for the disassembly of CT.

Both the ELISA and SPR disassembly assays found that holotoxin disassembly by PDI is less efficient for LT than CT. Complete CTdisassembly was recorded for the SPR assay, whereas only partial disassembly of CT occurred with the ELISA assay. We have previously noted this difference^20^ and attribute the greater extent of toxin disassembly in the SPR assay to technical differences between the two assays: the additional shear force provided by the SPR perfusion buffer likely facilitates PDI-driven toxin disassembly.

### Analysis of structural differences between CT and LT

**Figure 6:**
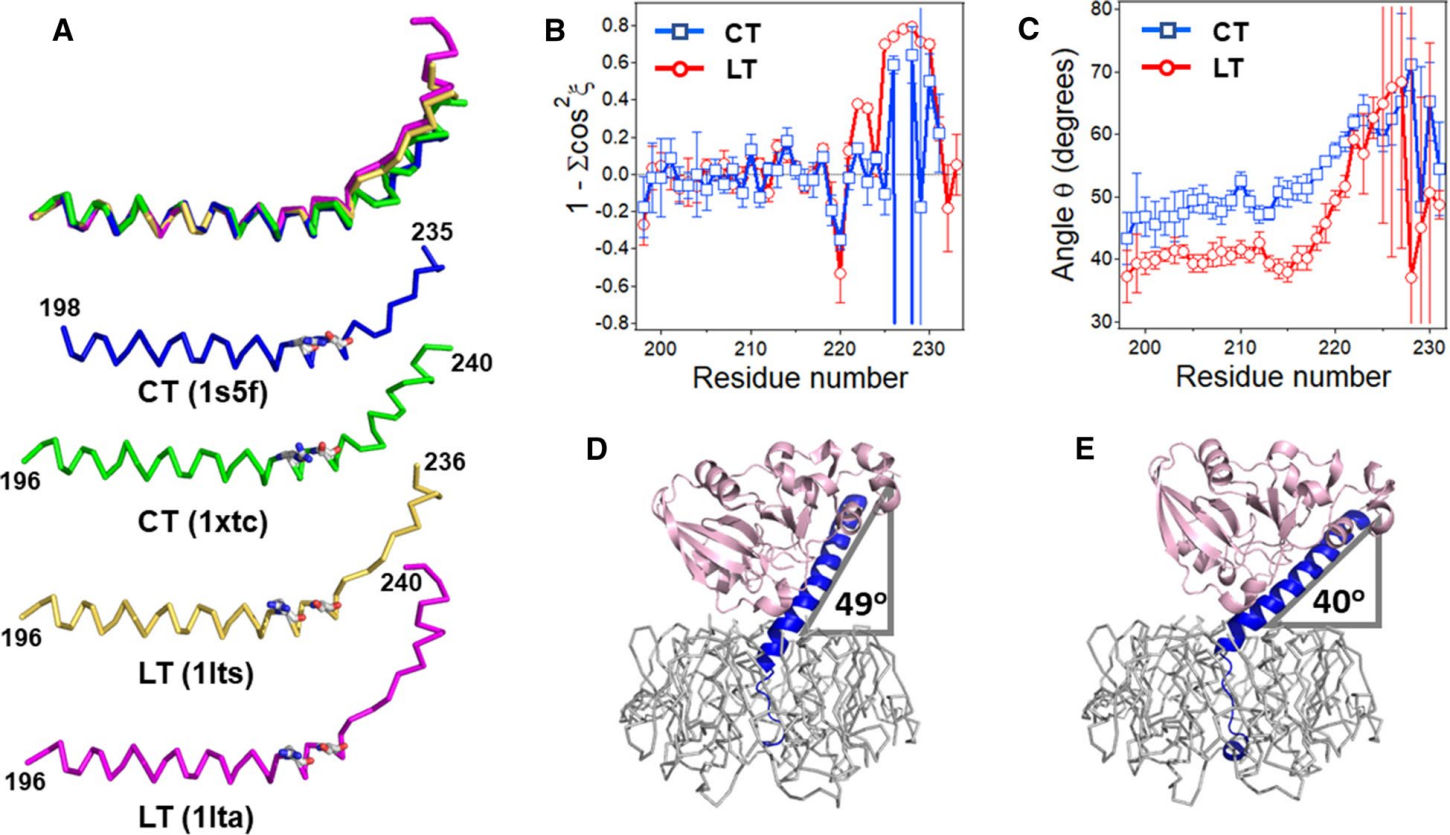
The A2 subunit orientation dictates the positioning of the A domain on the B homopentamer. (**A**) A2 subunits of two CT structures and two LT structures are shown superimposed (top) and separately, in ribbon format. In the top image, 1xtc, 1lts, and 1lta structures are aligned with the amino acid stretch 200–218 of 1s5f to highlight the structural differences in A2 tail region. N- and C-terminal residue numbers, as well as respective PDB entries are shown. Arg220 and Ser224 are presented in stick format colored according to element type (carbons grey, nitrogens blue, oxygens red). (**B**) Variation of 1 – (cos^2^*ξ*_1_ + cos^2^*ξ*_2_ + cos^2^*ξ*_3_) along the A2 chain identifies deviations from canonical α-helical structure of the A2 subunits. (**C**) Changes of the angle *θ* between the A2 helical axis and the B homopentamer plane along the A2 chain of CT and LT, as indicated. In (**B,C)**, average data for six CT and six LT structures (see Supplementary Table S1), with standard deviations, are shown. The maximum differences in *θ* were 2.6 degrees within the set of LT structures and 2.4 degrees within the set of CT structures. For each amino acid residue number *i*, the respective ordinate value applies to a quadruplet from *i* to *i* + 3. (**D**,**E**) The 1s5f structure of CT (**D**) and the 1lts structures of LT (**E**) are shown to highlight the average tilt angles of the A2 domain relative to the B_5_ domain. The A1 (pink) and A2 (blue) parts of the toxins are shown in cartoon format, and the B pentamers (grey) are shown in ribbon format. Images in (**A**,**D**,**E)** have been rendered by PyMOL 1.8.6.0 (Schrödinger, LLC).

**Figure 7:**
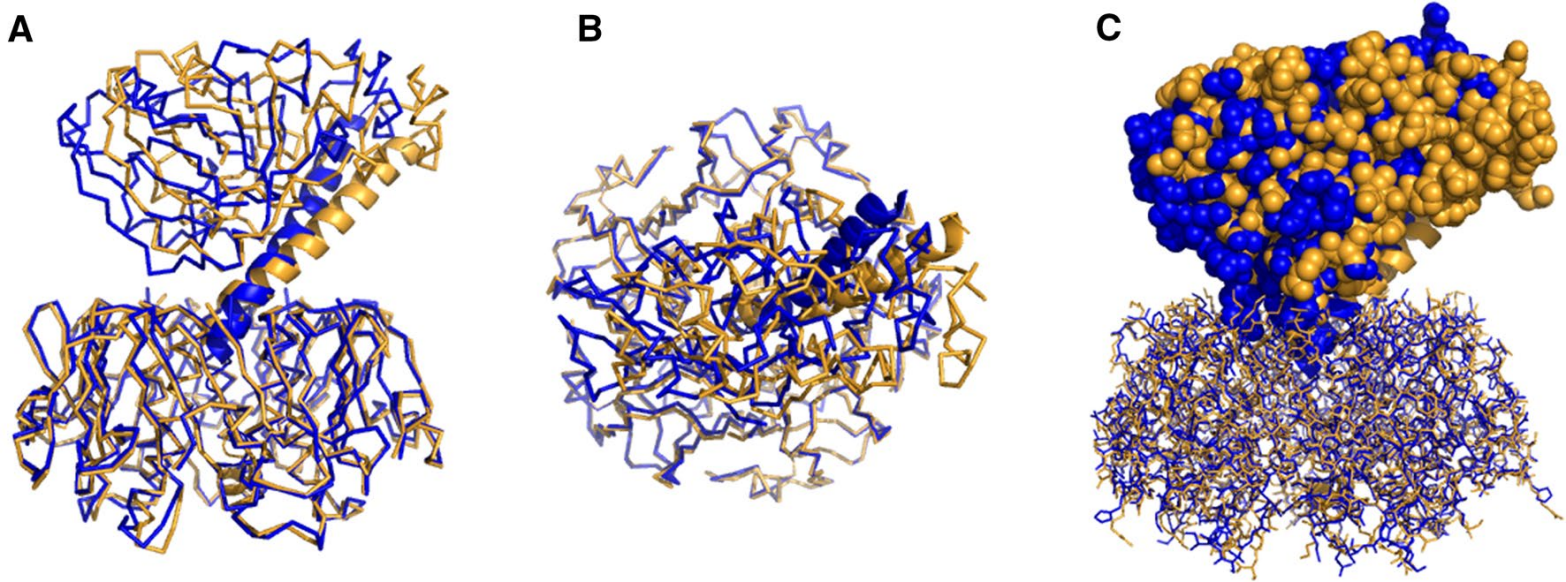
Superimposed structures of CT (blue) and LT (gold) (PDB entries 1s5f and 1lts, respectively). B pentamers of the two structures have been superimposed using the Align function of PyMOL to underscore the different A2 subunit orientations and distinct geometric positioning of the A1 subunits (**A,C**: side view, **B**: top-down view). Protein main chains in (**A**) and (**B**) are shown in ribbon format except for A2 domains, which are in cartoon format. In (**C**), the B pentamer is shown in “lines” format and the A1 and A2 chains in spheres and cartoon formats, respectively. Images are generated by PyMOL 1.8.6.0 (Schrödinger, LLC).

The higher purported stability of CT was originally explained by the helical structure of the A2 tail and its extensive direct contact with the B_5_ pore^13^, reported in the first X-ray crystal structure of CT (PDB ID 1xtc)^11^. However, later crystal structures of CT in various crystal forms^10^ reported a different, extended structure for the A2 tail, resembling that of LT more than the 1xtc structure of CT (Fig. 6A), practically invalidating this explanation. The geometry of the latter CT structures was also greatly improved in comparison to the original structure, as previously noted^1,23^. Here, we show by comparing crystal structures from different crystal environments (for details, see Supplementary Table S1 and Materials and methods) that the major, reliable structural difference between CT and LT is not the secondary structure of the A2 tail, but rather the larger angle between the A2 main α-helix and the B pentamer plane for CT compared to LT (Fig. 6). Furthermore, we show that this tertiary structural difference translates into a different positioning of the whole A domain with respect to the B pentamer (Fig. 7). This structural feature could result in distinct modes of PDI binding and disassembly of CT and LT, which, in turn, would play a major role in differential toxicities of these two otherwise structurally similar toxins.

The first step toward this goal was the assessment of the detailed geometry of the A2 subunit using an analytic geometry algorithm specifically developed for this purpose^24^. The α-helix was divided into overlapping quadruplets, i.e., 4 consecutive amino acid residues from 1 to 4, 2 to 5 and so forth, along the whole A2 chain. The three direction cosines (cos*ξ*) of the helical axis of each quadruplet were simulated using the C_α_ atom coordinates and the parameters for a canonical α-helix, e.g., 1.5 Å rise per residue along the helical axis. The dependence of the direction cosines on the amino acid residue numbers identified the degree of curvature of the helix, while the criterion 1 − ∑cos^2^*ξ*_i_ = 0 determined the local α-helical quality for overlapping 4-residue segments (Supplementary Fig. S2). Significant deviations of 1 − ∑cos^2^*ξ*_i_ from zero (by more than ± 0.2) indicate non-α-helical geometry. These data are summarized in Supplementary Fig. S2 for six LT structures and six CT structures, and the averaged values of 1 − ∑cos^2^*ξ*_i_ for LT and CT A2 chains are shown in Fig. 6B. The structures were selected to include wild-type toxins free of ligands and with ligands bound to the receptor-binding B pentamer, as well as toxins with point mutations in the A1 subunit, as detailed in Supplementary Table S1. For all structures, 1—∑cos^2^*ξ*_i_ displays significant deviations from zero starting at residue 220, indicating departure from α-helical geometry beyond that point (Supplementary Fig. S2 and Fig. 6B). (Note that data for residue *i* apply to a stretch from *i* to *i* + 3; see above.) This is consistent with the observation that a H-bonding between Arg220 carbonyl oxygen and Ser224 side chain contributes to the tilt of the A2 chain^5^ (Arg220 and Ser224 are highlighted in Fig. 6A). Also notable is that in no case the direction cosines are constant in the main helical region of A2 (Supplementary Fig. S2), indicating curved A2 α-helices for all structures. For LT structures, the A2 chain returns to a helical geometry for C-terminal residues 232–240 (Supplementary Fig. S2).

Next, the angle *θ* between the main helix of A2 and the B_5_ plane was simulated using the C_α_ atom coordinates (see Methods for details). Again, the angles were determined for overlapping quadruplets and plotted as a function of residue numbers (Fig. 6C). Tilt angles, averaged for six LT and six CT structures, show striking difference between A2 helix orientations in LT and CT structures, i.e. a significantly larger *θ* angle for CT than LT (Fig. 6C). Since the helical structure continues up to residue 221 (Fig. 6A), the angles of all amino acid quadruplets for each individual structure were averaged from residue 200 to 221, as summarized in Supplementary Table S1. The average of these average values was *θ* = 49.1^o^ ± 0.87° for CT (*θ* = 49.2^o^ ± 0.94^o^ without the poorly refined 1xtc structure) and *θ* = 40.5^o^ ± 0.93° for LT (*p* < 0.0001, Student's t test). Averaging for all 114 quadruplets of CT and those for LT yielded larger standard deviations: *θ* = 49.1^o^ ± 3.1° for CT and *θ* = 40.5^o^ ± 2.0 ^o^ for LT but *p* value in the same range (*p* < 0.0001, Student's t test), again documenting statistically significant difference between the A2 orientation for LT and CT, irrespective of crystal forms, ligands bound to the B pentamer, or mutations in the A1 chain. Also, the A2 helical tilt angle gradually increases between residues 200 and 213 for CT whereas it is relatively constant for LT (note that data at residue 210 apply to the quadruplet 210–213). This tertiary structural difference between LT and CT holotoxins is illustrated in Fig. 6D,E.

This 9-degree difference sustains along the A2 main helix, up to residue number 218. After that, the A2 helix undergoes significant bending in preparation to enter the B_5_ pore, more severe for LT than CT (Fig. 6C). The angle *θ* gradually increases to ~ 60° at residue numbers 224–225, then the simulations crash, producing meaningless values with very large deviations due to the loss of the helical structure, as indicated by strong divergence of 1 − ∑cos^2^*ξ*_i_ from zero (Fig. 6B).

As seen from these data, the important structural difference between CT and LT is not the secondary structure of the A2 tail but the overall tertiary structure of these toxins, namely, the orientation of the A2 subunit in relation to the B pentamer. Significantly different bending of the A2 chains of CT and LT results in distinct interdomain architectures of these toxins, i.e., specific positioning of the entire A subunit with respect to the B pentamer. These differences are highlighted in Fig. 7, which shows the CT and LT structures with superimposed B pentamers. The distinct angles of CTA2 (blue) and LTA2 (gold) in relation to the B pentamer are evident from the side view of the toxins (Fig. 7A), but differences can also be seen from a top-down view which reveals a more curved helix for the A2 subunit of CT than LT (Fig. 7B). The differing A2 architectures, in turn, shift the relative positions of the A1 subunits such that LTA1 is more strongly tilted toward its B pentamer than CTA1, resulting in a wider A1/B_5_ cleft for LT than CT (cf. Figure 1 and Fig. 7C). These crucial structural features could determine special, distinct modes of PDI binding to the toxin, as shown in the next section.

### Analysis of PDI-toxin interaction by protein docking simulations

**Figure 8:**
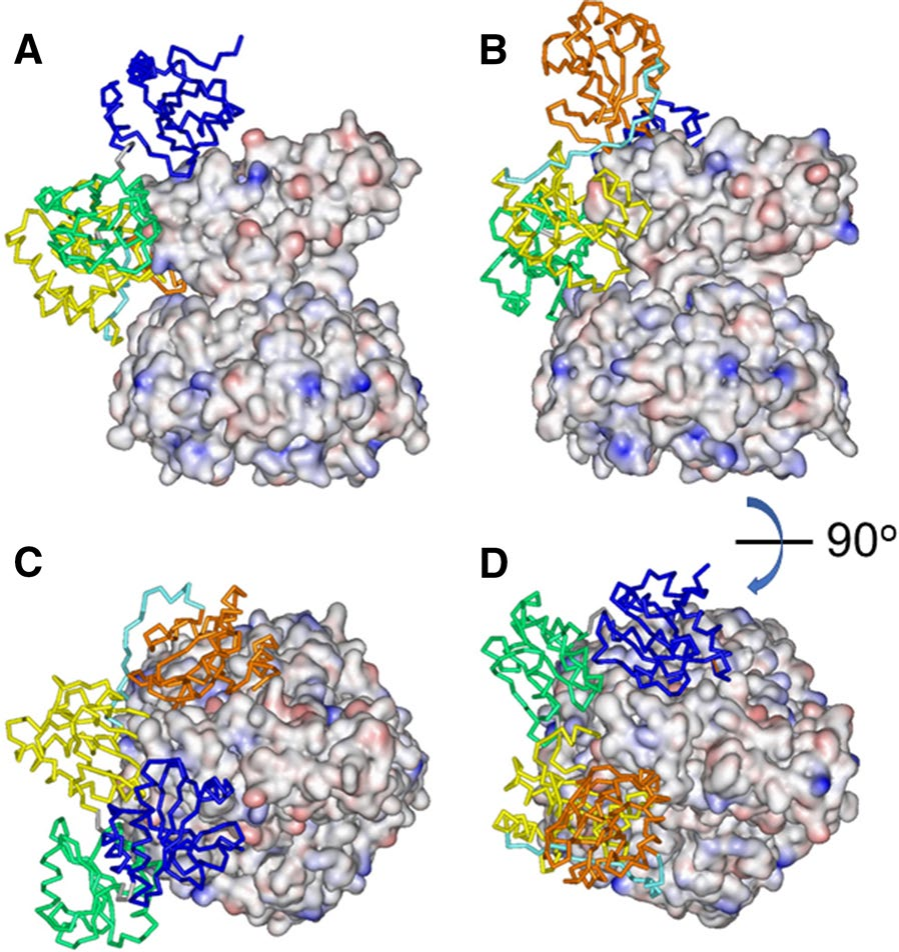
Models for interaction of reduced PDI, shown in ribbon format, with CT (**A**,**C**) and LT (**B**,**D**), shown in electrostatic surface format. (**A**,**B**) The toxins are presented in side-view orientation. (**C**,**D**) The structures are rotated about a horizontal axis by 90° to generate top views. Toxin surface coloring reflects the electrostatic charge: blue = positive charge, red = negative charge, white = neutral. PDI coloring is domain-specific: **a** = blue; **b** = green, **b′** = yellow, **x** = cyan, **a′** = orange (from N- to C-terminus). The structures of CT, LT, and PDI are derived from PDB entries 1s5f, 1lts, and 4ekz, respectively. Images have been rendered by Viewer Lite 4.2.

The next step was to analyze whether PDI binds to CT and LT in different modes, which could contribute to more efficient disassembly of CT by PDI compared to LT. PDI-toxin docking data were obtained using the ClusPro server. The reduced structure of PDI (PDB entry 4ekz)^25^ was used for this analysis because only reduced PDI interacts with CT^17,18^. For each pair (i.e., PDI-CT and PDI-LT), ClusPro provided 30 PDI-toxin structures. We have shown earlier that the functional PDI-toxin interaction involves the A1 subunit of CT, and that the major PDI binding motif centers on the A1-1 subdomain of CTA1 (i.e., the first 133 residues of the subunit)^18^. Based on this criterion, docked complexes involving PDI binding to the C-terminal region of the A1 subunit or the B pentamer were dismissed as they were biologically irrelevant. Two structures for PDI-LT and one structure for PDI-CT displayed extensive interaction between PDI and the A1-1 subdomain of the A1 chain (Fig. 8A,B). However, there were two major structural differences between the PDI-CT and PDI-LT complexes: (i) PDI orientation when bound to CT was antiparallel compared to its structure when bound to LT (Fig. 8C,D), and (ii) PDI fit into the A1/B_5_ cleft of LT better that that of CT. The latter feature is expected, as the tertiary structure of LT provides a ~ 9° wider A1/B_5_ cleft for PDI binding compared to CT (see above), but the antiparallel binding of PDI to the two toxins is an unexpected result. The important finding from these docking simulations, however, is the suggestion of significantly different modes of PDI-toxin interaction for CT and LT, which may have important functional implications, as discussed below.

## Discussion

CT and LT are highly related AB_5_-type protein toxins with the same GM1 surface receptor, intracellular trafficking/translocation itineraries, and cytosolic Gsα target^1,2^. The toxins also exhibit identical kinetics of reduction by DTT or GSH (Supplementary Fig. S3). Despite these striking similarities, CT is more potent than LT^12,13^. This difference has been attributed to the non-catalytic A2 subunit^13^, but the underlying molecular mechanism remains unknown. The original hypothesis that the A2 tail of CT provides a stronger interaction with the B pentamer and thereby imparts a higher stability and toxicity to CT^13,14^ is no longer valid because (i) later, improved CT structures show an A2 tail structure similar to that of LT (Fig. 6A,D,E) and (ii) direct examination of native CT and native LT did not detect any difference in toxin stability (Fig. 3). Other factors, including interactions with host chaperones, must be taken into consideration.

Our approach focused on the overall 3-dimensional structures of CT and LT and their molecular interactions with PDI, a key chaperone responsible for displacing the catalytic A1 subunit from the rest of the toxin. This disassembly event is required for cellular activation of the toxins, which explains why PDI-deficient cells are completely resistant to CT^18^ and LT (manuscript in preparation). Cell-based assays, ELISA measurements, and an SPR system identified correlations between CT and LT toxicities and PDI-mediated disassembly of the toxins (Figs. 2, 4, 5). Molecular simulations predict that distinct positioning of the CT and LT A domains determines PDI binding in unique configurations. PDI appears to fit into the wider A1/B_5_ cleft of LT with relative ease, except for the **a′** domain, whereas in the case of CT the whole **a** domain hangs outside of the cleft (Fig. 8). It should be emphasized that the structures generated by ClusPro are based on rigid-body fitting, whereas these proteins, especially PDI^26,27^, are flexible macromolecules rather than rigid bodies. Thus, the structures of the PDI-CT and PDI-LT complexes shown in Fig. 8 should be considered as starting points and combined with the earlier finding that PDI partially unfolds and expands upon binding to the A1 subunit of CT^19^. Expansion of PDI in a tighter A1/B_5_ cleft of CT might therefore exert a stronger dislodging force than in the case of LT, resulting in a more efficient disassembly of CT.

The significant sequence difference at the C-terminal region of the CT and LT A2 subunits is likely important for PDI-assisted toxin disassembly, but not because of local secondary structural dissimilarities. Instead, the sequence difference in the boundary of A2 helix and A2 tail determines different tilts of the A2 chain, which positions the whole A domains of the toxins at specific orientations. It is interesting to note that the tilt of LTA2 has been ascribed to H-bonding between Arg220 main chain carbonyl group and Ser224 side chain^5^ (1lts). This interaction apparently also contributes to disruption of the A2 helical structure. However, the Arg220-Ser224 H-bonding is present in all twelve LT and CT structures examined in this work (see Fig. 6A for some examples). The average carbonyl-to-hydroxyl distance is 2.63 ± 0.14 Å for six CT structures and 2.53 ± 0.18 Å for six LT structures, implying the difference in A2 orientation cannot be explained by this H-bonding. The kink in the A2 chain following residue 220 echoes with an interesting observation that LT structures in different crystal forms exhibit distinct hinge-like rotations of the A2 chain, and hence the whole A subunit, around the residue Gln221, deemed to be caused by crystal contacts^4,28^. The rotation is between 2 and 7 degrees but does not occur about a certain axis, and therefore is related to the tilt angle *θ* discussed in this work only partially. In fact, maximum differences in *θ *within LT structures is 2.6 degrees and that within CT structures is 2.4 degrees (Supplementary Table S1). These findings indicate that the statistically significant difference in positioning of the A subunit with respect to the B pentamer for LT and CT, reported in this work, could not result from different crystal contacts, as both groups include structures of different crystal forms.

Although Arg220-Ser224 H-bonding cannot explain the different A2 tilt angles for CT and LT, Craft, Jr. et al. (2015) have noted that amino acid residue 229 in the A2 subunit helps position the A2 α-helix with respect to the B pentmaer^14^. This residue is located near the A2 bend and represents one of the four amino acid differences between CT and LT in the region of the A2 subunit that determines toxin potency (red ball and sticks in Fig. 1). It is therefore possible that this residue, either alone or in combination with the other three differing residues (230, 232, and 233), alters the tilt angle of the A2 chain. This would result in distinct orientations of the A2 helix relative to the B pentamer (Fig. 6), producing different tertiary structures for CT and LT (Fig. 7) that could impact the mode of PDI docking (Fig. 8), the efficiency of PDI-driven toxin disassembly (Figs. 4–5), and, ultimately, toxin potency (Fig. 2). However, causative links between A2 helix orientation, PDI-driven toxin disassembly, and toxin potency remain to be established. Future work to examine those links can use CT or LT mutants with one or more altered residues at positions 229, 230, 232, and 233 to potentially alter the A2 tilt angle. Mutants with the A2 tilt angle of wild-type CT are predicted to exhibit efficient PDI-driven toxin disassembly and high potency, while mutants with the A2 tilt angle of wild-type LT should exhibit inefficient PDI-driven toxin disassembly and low potency. Inefficient disassembly of the H2 hybrid toxin by PDI and its low cellular activity (Supplementary Fig. S4) provide preliminary support for this model, but the A2 tilt angle in this chimeric toxin—as well as any other mutant toxin designed to test the model—needs to be determined by X-ray crystallography for more definitive data analysis.

In conclusion, data presented in this work report that (i) CT and LT do not differ in stability, which was the previous explanation for their different potencies; (ii) the PDI-driven disassembly of CT is more efficient than its disassembly of LT; (iii) there are previously unrecognized differences in the A2 tilt angles for CT and LT that influence the orientation of the their A1 subunits in relation to the B pentamer; and (iv) those different orientations are predicted, by molecular simulations, to alter the docking of PDI to CT vs. LT. These collective results form the basis for a new model of toxin potency in which the unique interdomain arrangements of CT and LT determine their differential toxicity through specific interactions with PDI.

## Materials and methods

### Materials

Ganglioside GM1 and PDI were purchased from Sigma-Aldrich (St. Louis, MO). Trypsin, soybean trypsin inhibitor, and TMB were purchased from Fisher Thermo Scientific (Waltham, MA). A rabbit antibody against CTA1 was purchased from Advanced Targeting Systems (Carlsbad, CA), a goat antibody against CTB was purchased from Cayman Chemicals (Ann Arbor, MI), and a HRP-labeled goat anti-rabbit IgG antibody was purchased from Jackson ImmunoResearch (West Grove, PA).

### Plasmids, site-directed mutagenesis and cloning

Vector pARCT5, a gift from Dr. Randall K. Holmes (University of Colorado School of Medicine, Aurora, CO), contains an arabinose-inducible CT operon with signal sequences derived from the LT-IIb B gene^29^.

For recombinant expression of LT from a human enterotoxigenic *E. coli* strain (PE0415, elt operon, GenBank: EU113247.1^30^), we designed vector pARhLT5 based on pARCT5, but with DNA coding for LTA and LTB replacing CTA and CTB (signal sequences from the LT-IIb B gene remain unchanged). The DNA was synthesized by Genscript (hLT-pUC57) and cloned into pARCT5, in which an NcoI restriction site (in the *chloramphenicol* gene) was removed by a silent mutation (Q5^®^ Site-Directed Mutagenesis Kit, New England Biolabs, Ipswich, MA) using primers Mut 1 and 2 (Supplementary Table S2) and an annealing temperature of 64 °C. Mutated pARCT5 was linearized with NcoI (FastDigest, Fisher Thermo Scientific). The gel-purified vector (QIAquick Gel Extraction Kit, Qiagen, Hilden, Germany) was treated with rSAP (New England Biolabs), before inserting the hLT DNA fragment using T4 DNA Ligase (Thermo Scientific). pARhLT5 was sequenced (GATC Biotech, Konstanz, Germany) using primers Seq1–3 (Supplementary Table S2).

To clone the H1 hybrid toxin (CTA/LTB chimera), plasmids pARCT5 and pARCT5hLT were digested with NheI and HindIII (Thermo FastDigest). The pARCT5-derived vector (8184 bp) and the pARCT5hLT-derived insert (1316 bp) were gel purified and ligated using T4 DNA Ligase. The resulting plasmid pARCTALTB was sequenced using primers Seq1, 4 and 5 (Supplementary Table S2). An analytical restriction digest with NcoI resulted in the expected bands on a gel.

To generate the H2 hybrid toxin (CTA(EVDIY)/LTB chimera), site-directed mutagenesis (Q5® Site-Directed Mutagenesis Kit, New England Biolabs) was performed using pARCTALTB, primers Mut 3 and 4 (Supplementary Table S2), and an annealing temperature of 59 °C. Successful mutagenesis was verified by Sanger sequencing using primers 1, 4 and 5 (Supplementary Table S2).

### Expression and purification of holotoxins

Protein expression and purification of holotoxins was performed as described previously^31^. Briefly, the genes for human LT, CT, H1 hybrid toxin, and H2 hybrid toxin were expressed in OverExpress™ C43 (DE3) cells (Sigma-Aldrich). Cells were grown at 37 °C in TB medium containing chloramphenicol until an OD_600 nm_ of 2.0 was reached. Cells were then induced with l-arabinose and harvested after 3 h. Holotoxins were extracted from the bacterial pellet by inducing periplasmic lysis with polymyxin B sulfate (Sigma-Aldrich). Holotoxins were purified by TALON affinity chromatography using a HiTrap TALON crude column (GE Healthcare, Chicago, IL) and size exclusion chromatography with a HiLoad 16/60 Superdex 200 prep grade column (GE Healthcare) equilibrated with phosphate-buffered saline. To reduce B-pentamer contamination due to partially overlapping peaks, only size exclusion fractions prior to the holotoxin peak maximum were pooled, filtered and stored at 4 °C. The hybrid toxins were characterized by SDS-PAGE analysis, tryptic digestion and mass spectrometry.

### Toxicity assay

Before use, holotoxins at 1 µg/mL concentrations were nicked with 1 µg/mL of trypsin for 30 min at 25 °C. Trypsin inhibitor (2 µg/mL) was then added to the toxin. As previously described^18^, CHO cells were incubated with nicked toxin for 2 h before an ELISA-based kit (GE Healthcare) was used to quantify intracellular cAMP. Basal cAMP levels from unintoxicated cells were also calculated and subtracted from the values for toxin-treated cells. The data were expressed as percentages of the maximal cAMP response for the experiment, which was generated by 100 ng/mL of CT, corresponding to 1.23 nM.

### ELISA assays

GM1 (6 µg/mL in ethanol) added in 50 µL volume to the wells of a 96-well plate was allowed to air dry overnight at room temperature. The plates were then washed four times with 100 µL of phosphate-buffered saline (pH 7.4) containing 0.05% Tween 20 (PBS-T) before adding 100 µL of toxin at 1 µg/mL in PBS-T with 2.5% bovine serum albumin (BSA) or, for the background control, PBS-T with 2.5% BSA alone. After 1 h at 4 °C, all wells were washed four times with PBS-T to remove unbound toxin from the plate. The prepared plates were then used immediately.

For assays of toxin stability, the toxin-coated wells were exposed to McIlvaine buffer (pH 5.5) ± 0.1% SDS for 30 min at 25 °C. This was followed by four PBS-T washes, incubation with a rabbit anti-CTA1 primary antibody (100 µL at 1:1000 dilution) for 1 h at 4 °C, and incubation with an HRP-conjugated goat anti-rabbit IgG antibody (100 µL at 1:1000 dilution) for 30 min at 4 °C. TMB substrate was added for 5–10 min before addition of a stop solution and absorbance measurement at 450 nm with a Synergy 2 plate reader (BioTek, Winooski, VT). The percentage of toxin disassembly was then calculated as [1.00 − (treated A1 signal/untreated control A1 signal)] × 100.

For assays of PDI-driven toxin disassembly, PDI (20 µg/mL) was pre-reduced with 1 mM DTT for 30 min at 25 °C. The toxin-coated wells were then incubated with 100 µL of PDI (2 µg with 1 mM DTT still present) for 1 h at 37 °C before antibody processing and calculations of toxin disassembly as described above for the stability assay. To establish the untreated A1 control signal, toxin-coated wells were exposed to 1 mM DTT in the absence of PDI. Previous studies have shown that reduction alone is not sufficient for toxin disassembly^16–18^.

### SPR-based toxin disassembly assay

Experiments were performed using a Nicoya (Kitchener, ON, Canada) OpenSPR instrument. The flow rate was 20 µL/min for all steps other than PDI injection, which was performed at 40 µL/min. Volumes of 250 µL were used for each injection, and all samples were placed in 10 mM MES (pH 7.4) with 200 mM NaCl. GM1 (150 µg/mL) was immobilized on a gold hydrophobic sensor with OGP surface activation. This was followed by two injections of PBS with 2.5% BSA. Nicked and reduced toxin (CT or LT, 10 µg/mL) was then added to the GM1-coated sensor. After another injection of PBS/BSA, pre-reduced PDI (1 or 10 µM in buffer containing 1 mM DTT) was added to the sensor through a 150 s injection and 250 s chase for signal stabilization. An anti-CTA1 antibody (1:1,000 dilution) was then added to the sensor, which was followed by injection of an anti-CTB antibody (1:1000 dilution). Each experiment used a fresh sensor; no sensors were regenerated after the antibody additions.

CT and LT were nicked and reduced through a 30 min 4 °C incubation in buffer containing 1 mM DTT and 10 µg/mL of trypsin. Soybean trypsin inhibitor (20 µg/mL) was then added for another 30 min before toxin injection. PDI was reduced with 1 mM DTT (final concentration) for 30 min at 25 °C before injection. No loss of signal occurred when CT or LT was exposed to DTT-containing buffer in the absence of PDI, which was consistent with previous reports on the stability of reduced CT^16–18^.

### Analysis of protein interdomain orientation

The orientation of the helical axis for the A2 subunit was simulated using the HELO (HELix Orientation) algorithm^24^. Briefly, the C_α_ atom coordinates of A2 were used to calculate the direction cosines, i.e., the cosines of angles between the helical axis for four consecutive amino acid residues and the *X*, *Y*, and *Z* axes of the intrinsic coordinate system of the protein, cos*ξ*_1_, cos*ξ*_2_, cos*ξ*_3_, respectively These cosines were simulated for overlapping quadruplets along the entire A2 subunit, i.e., for residues 1–4, 2–5, 3–6…, which provided the local helical orientations along the A2 chain and allowed determination of the average orientation for any helical stretch.

Novel analytic geometry simulations were used to quantitatively describe the orientation of the A2 subunit relative to the CT or LT protein molecules. This goal was achieved by determining the angle between the A2 helical axis and the plane of the B_5_ ring, i.e., a plane made by the C_α_ atoms of identical amino acids in five units of the B pentamer. We chose Leu72 as such amino acid because it is located in the middle of a stable α-helix in each of B_5_ units of both CT and LT. In 3-dimensional space, a plane can be defined by three points, whereas we actually had five points, i.e., the C_α_ atoms of five Leu72 residues. These five points were arranged in ten (maximum possible number) triplets, and the orientations of ten planes were simulated as follows. For each triplet, the nine coordinates (*x*, *y*, and *z* coordinates of three C_α_ atoms) were used to define a plane:

1 $$\left\{\begin{array}{c}{x}_{1}+B{y}_{1}+C{z}_{1}+D=0\\ {x}_{2}+B{y}_{2}+C{z}_{2}+D=0\\ {x}_{3}+B{y}_{3}+C{z}_{3}+D=0\end{array}\right.$$

These equations were solved together to find the coefficients B, C, and D:

2 $$B= \frac{\left({x}_{3}-{x}_{1}\right)\left({z}_{2}-{z}_{3}\right)+\left({x}_{2}-{x}_{3}\right)\left({z}_{1}-{z}_{3}\right)}{\left({y}_{1}-{y}_{3}\right)\left({z}_{2}-{z}_{3}\right)+\left({y}_{3}-{y}_{2}\right)\left({z}_{1}-{z}_{3}\right)}$$

3 $$C=\frac{{x}_{3}-{x}_{2}+B\left({y}_{3}-{y}_{2}\right)}{{z}_{2}-{z}_{3}}$$

4 $$D=-{x}_{3}-B{y}_{3}-C{z}_{3}$$

The angles *α*, *β*, and *γ* between the plane normal and the *X*, *Y* and *Z* axes of the protein coordinate system were determined as:

5 $$\alpha ={cos}^{-1}\left(\frac{1}{\sqrt{1+{B}^{2}+{C}^{2}}}\right)$$

6 $$\beta ={cos}^{-1}\left(\frac{B}{\sqrt{1+{B}^{2}+{C}^{2}}}\right)$$

7 $$\gamma ={cos}^{-1}\left(\frac{C}{\sqrt{1+{B}^{2}+{C}^{2}}}\right)$$

The angles were averaged from ten simulations to determine the mean orientation of the B pentamer normal. The angle between the A2 helical axis and the B pentamer normal, *δ*, was then calculated as follows:

8 $$cos\delta =\frac{Aa+Bb+Cc}{\sqrt{\left({A}^{2}+{B}^{2}+{C}^{2}\right)\left({a}^{2}+{b}^{2}+{c}^{2}\right)}}$$

where *A* ≡ 1/cos*β*cos*γ*, *B* ≡ 1/cos*α*cos*γ*, *C* ≡ 1/cos*α*cos*β*, *a* ≡ 1/cos*ξ*_2_cos*ξ*_3_, *b* ≡ 1/cos*ξ*_1_cos*ξ*_3_, *c* ≡ 1/cos*ξ*_1_cos*ξ*_2_. Determination of angle *δ* for each quadruplet of the A2 domain revealed the variation of the helix orientation along the chain, i.e. the helical axis curvature. Moreover, application of the rule of direction cosines indicated deviations of local structures from canonical α-helical geometry. The angle *θ* between the A2 helix and the B pentamer plane was simply *θ* = 90° – *δ*.

Six LT and six CT structures were analyzed, as summarized in Supplementary Table S1. LT structures range from 1.95 Å resolution to 2.4 Å resolution and are either uncomplexed or in complex with Gal, lactose or T-antigen. 1lt4 corresponds to the S63K variant (A-subunit) and 1ltg to the R7K variant (A-subunit). All structures have space group *P*2_1_2_1_2_1_, but 1lta has greatly different cell parameters. CT structures belong to different crystal forms (*P*2_1_ with 3 different sets of unit cell parameters and *P*2_1_2_1_2_1_). Resolution ranges from 1.75 to 2.6 Å. Three structures are in complex with Gal, three are for Y30S variants (in A1-chain, not too far from subunit interface). 1s5d has Gal and Y30S. 1s5e has 2 molecules in the asymmetric unit.

### Protein–protein docking

Protein docking simulations were conducted using an internet-based server ClusPro^32–34^. Briefly, the procedure involves rigid body docking using fast Fourier transform correlation, root mean square deviation-based identification of 1000 top-ranking complexes based on energy minimization, correction of steric conflicts and refinement of best, most reliable complexes.

## Supplementary Information

Supplementary Information.
