# Supplementary material for: Holotoxin disassembly by protein disulfide isomerase is less efficient for Escherichia coli heat-labile enterotoxin than cholera toxin
Source: Sci Rep. 2022 Jan 7;12:34. doi: 10.1038/s41598-021-03939-9 (PMC8741891; doi:10.1038/s41598-021-03939-9)
Supplement: Supplementary file 1 — Supplementary Information. [file 41598_2021_3939_MOESM1_ESM.pdf]

Supplementary Information

Holotoxin disassembly by protein disulfide isomerase is less efficient for *Escherichia coli* heat-labile enterotoxin than cholera toxin

Albert Serrano[1,*], Jessica L. Guyette[1,*,‖], Joel B. Heim[2], Michael Taylor[1], Patrick Cherubin[1,¶], Ute Krengel[2], Ken Teter[1,#], and Suren A. Tatulian[3,#]

[1]Burnett School of Biomedical Sciences, College of Medicine, University of Central Florida, Orlando, FL 32816

[2]Department of Chemistry, University of Oslo, Blindern, Norway.

[3]Department of Physics, College of Sciences, University of Central Florida, Orlando, FL 32816

[*]These authors contributed equally to the work

[#]email: statulia@ucf.edu or Kenneth.Teter@ucf.edu

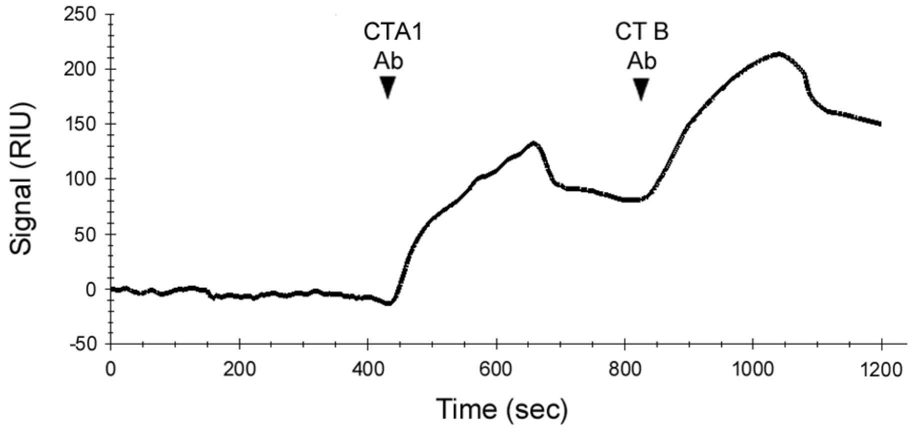

Figure S1. CT disassembly is not triggered by DTT alone. Trypsin-nicked CT was appended to a GM1-coated SPR sensor, and a baseline measurement corresponding to the mass of the sensor-bound holotoxin was taken to establish the 0 RIU signal. Buffer containing 1 mM DTT in the absence of PDI was added to the sensor at 0 sec, followed by sequential injections of antibodies against CTA1 and CTB as indicated. The consistent 0 RIU signal, combined with the positive signals for CTA1 and CTB antibodies, indicated that reduced CT remained on the plate as an intact holotoxin for the duration of the experiment. The lack of CT disassembly following reduction is consistent with previous reports[1-3].

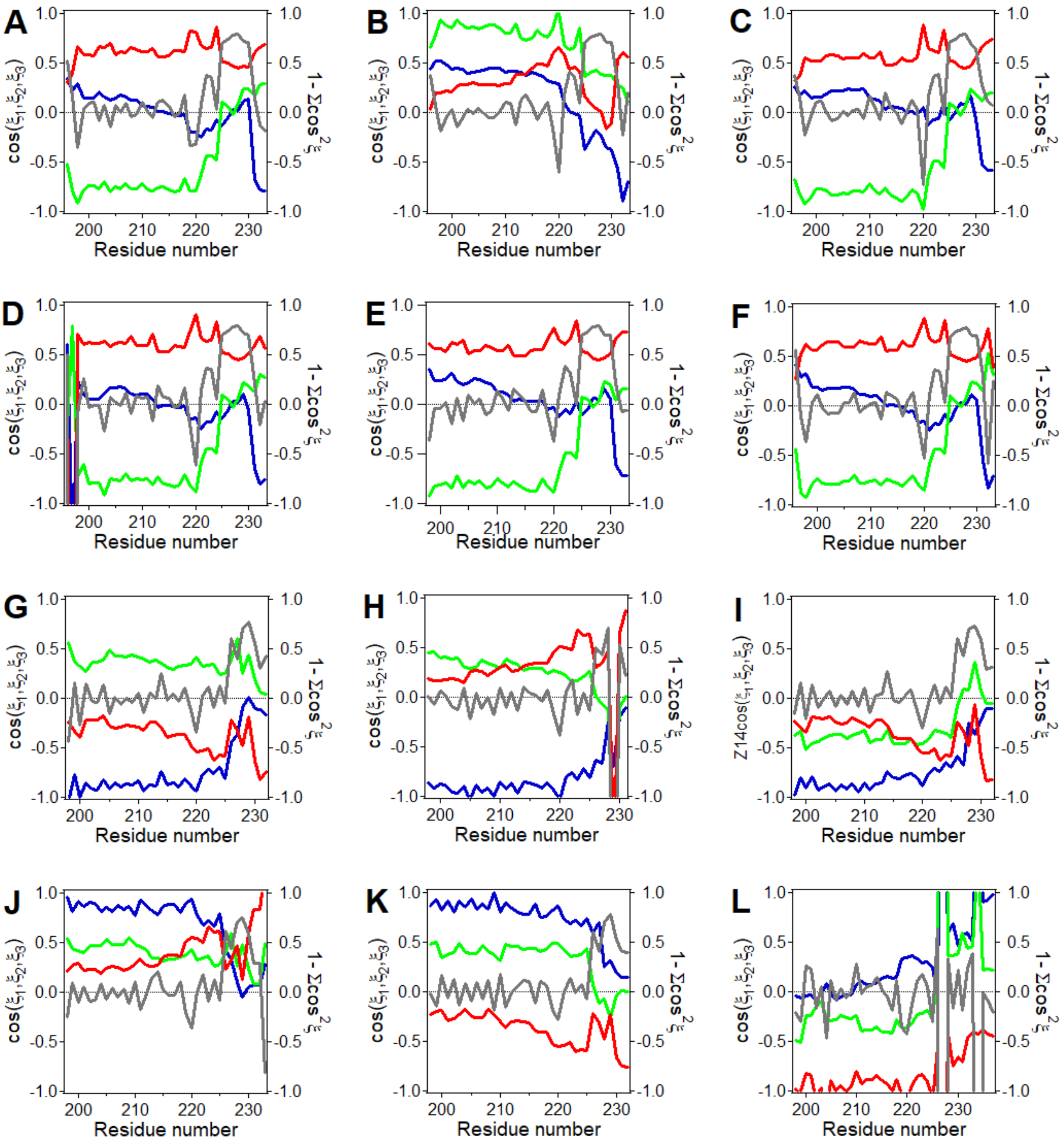

Figure S2. Direction cosines and $1 - \Sigma\cos^2\xi$ for A2 helical axis of LT and CT. (**A-F**) LT (PDB IDs 1lt4, 1lta, 1ltg, 1lti, 1lts, 1ltt, respectively). (**G-L**): CT (PDB IDs 1s5b, 1s5c, 1s5d, 1s5e, 1s5f, 1xtc, respectively). Color code: $\cos(\xi_1)$, $\cos(\xi_2)$, and $\cos(\xi_3)$ are shown in red, green, and blue, respectively, and $1 - \Sigma\cos^2\xi$ is shown in grey. (ξ_1, ξ_2, and ξ_3 are the angles between the A2 helical axis and the *X*, *Y*, and *Z* axes of the protein coordinate system.)

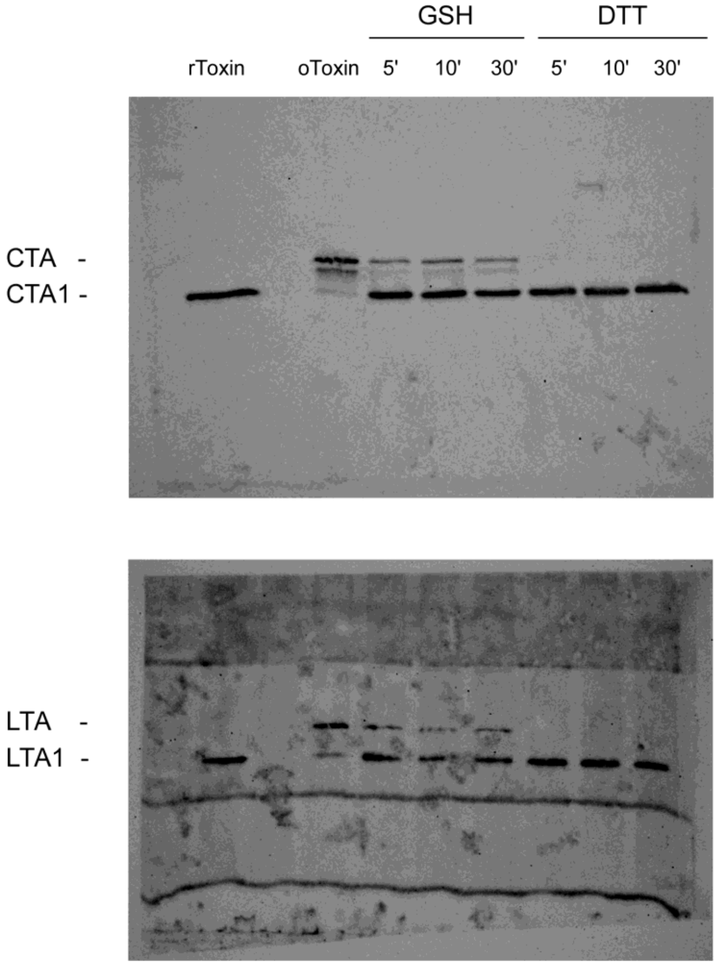

Figure S3. Kinetics of CT and LT reduction. Trypsin-nicked samples of CT and LT were incubated at room temperature with 1 mM GSH or 1 mM DTT for 5, 10, or 30 min, as indicated. They were then placed in non-reducing Laemmli sample buffer, boiled immediately, and resolved by SDS-PAGE with 15% polyacrylamide gels. Western blot with a rabbit anti-CTA1 primary antibody and HRP-conjugated goat anti-rabbit IgG secondary antibody was used to visualize the oxidized A subunit (i.e., the disulfide-linked A1/A2 heterodimer) and the reduced, free A1 subunit. Oxidized toxin (oToxin) and toxin reduced with β-mercaptoethanol (rToxin) were loaded for reference. The A subunit is 26 kDa, and A1 is 21 kDa. One of two representative experiments is shown.

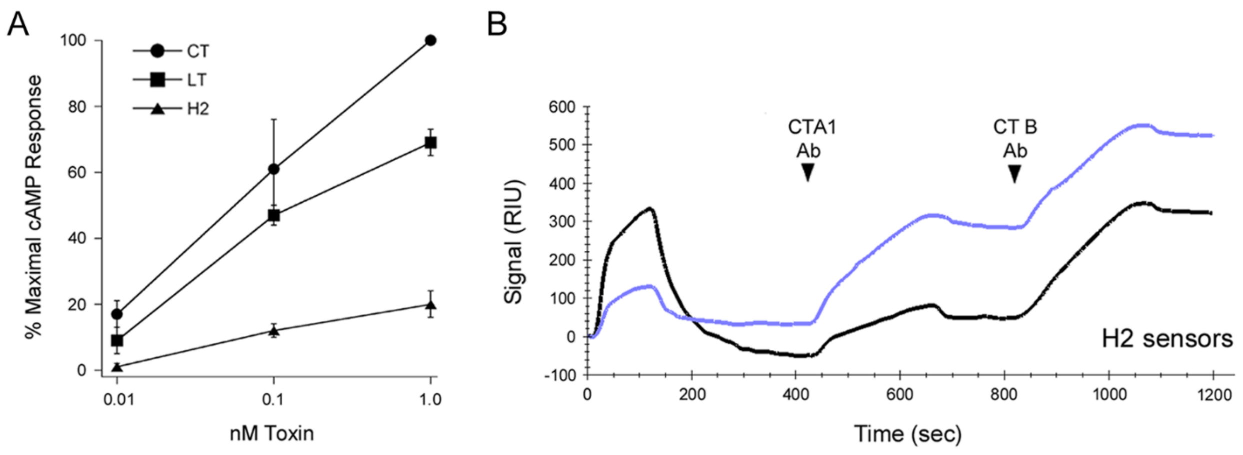

Figure S4. The H2 hybrid toxin exhibits low cellular activity and poor disassembly by PDI. The H2 hybrid toxin consists of the CTA1 subunit, a mutant CTA2 subunit with the LTA2 sequence between residues 226-236, and the LTB pentamer. **(A)** CHO cells were incubated for 2 h with the stated concentrations of CT, LT, or H2 before cAMP levels were quantified. Data are expressed as percentages of the maximum cAMP response for the assay (i.e., exposure to 1 nM CT) and are presented as average ± standard deviation of three independent experiments, each with triplicate samples. Rodighiero et al.[4] previously compared the potencies of CT and H2, but the data presented here represent the first direct comparison between the cellular activities of LT and H2. **(B)** Trypsin-nicked H2 toxin was appended to a GM1-coated SPR sensor. A baseline measurement corresponding to the mass of the bound holotoxin was recorded and set at 0 RIU. Buffer containing 1 mM DTT and either 1 µM PDI (blue) or 10 µM PDI (black) was then added to the sensor. This was followed by sequential injections of antibodies against the A1 and B subunits, as indicated. Traces for separate experiments with 1 µM and 10 µM PDI are overlaid here for comparative purposes. No disassembly occurred in the presence of 1 µM PDI. Only a fraction of H2 was disassembled by 10 µM PDI, as indicated by the minor drop in signal below the initial baseline value of the holotoxin (representing slight loss of the A1 subunit) along with a positive signal for the A1 antibody. In contrast, complete disassembly of LT occurred in the presence of 10 µM PDI (Fig. 5F of main text). The H2 hybrid thus exhibited both lower cellular activity and less efficient disassembly than LT, which was consistent with our model that predicts toxin potency is directly linked to the efficiency of PDI-driven toxin disassembly. We did not run similar experiments with the H1 hybrid because of its documented difference from both CT and LT in the stability assay shown in Figure 3 of main text.

Table S1. Angle θ between A2 α-helical axis and B pentamer plane for six CT and six LT structures [a]

Toxin	PDB ID	Maximum resolution	Mutation	Bound ligand	$\theta \pm$ SD (degrees)
CT	1s5e [5]	1.90 Å	none	5 Galactose [b]	49.2 ± 1.9
CT	1s5f [5]	2.60 Å	none	5 Galactose [b]	49.8 ± 2.8
CT	1s5b [5]	2.13 Å	A1 chain Y30S [c]	none [d]	50.0 ± 2.9
CT	1s5c [5]	2.50 Å	A1 chain Y30S [c]	none	47.6 ± 4.3
CT	1s5d [5]	1.75 Å	A1 chain Y30S [c]	5 Galactose [b]	49.2 ± 2.8
CT	1xtc [6]	2.40 Å	none	none	48.5 ± 2.8
LT	1lts [7]	1.95 Å	none	none	39.4 ± 2.1
LT	1lta [8]	2.20 Å	none	5 Galactose [b]	40.0 ± 1.4
LT	1ltt [9]	2.30 Å	none	5 Lactose [b]	40.6 ± 1.7
LT	1lti [10]	2.13 Å	none	1 T-antigen disaccharide [e]	42.0 ± 2.0
LT	1lt4 [11]	2.00 Å	A1 chain S63K [f]	5 Lactose [b]	41.0 ± 1.8
LT	1ltg [12]	2.40 Å	A1 chain R7K [g]	none	39.8 ± 1.9

[a] Average values ± SD for 19 overlapping quadruplets (displaced by 1 amino acid residue) between residues 200 and 221 of A2 chain. Wild-type CT forms two crystal forms, and the Y30S mutant forms three crystal forms[5].

[b] Crystallized in the presence of ligand; 1 ligand bound to the ventral side of each B subunit

[c] A1 subunit Y30S substitution imparts activity without the need of nicking/reduction.

[d] Crystallized in the presence of kemptide (artificial heptapeptide mimicking substrate for ADP ribosylation), but not seen in the electron density map.

[e] Crystallized in lactose-containing buffer, then soaked in lactose-free buffer and then in T-antigen tumor marker disaccharide (D-Gal-β1-3-GalNAc)-containing buffer. Binding of T-antigen disaccharide is expected as it is identical to the terminal sugar moieties of CT and LT receptor GM1 ganglioside. Only one disaccharide bound to a B subunit could be clearly seen, and galactose moieties were identified bound to three other B subunits[13].

[f] A1 subunit S63K substitution abolishes enzymatic activity of CT and LT.

[g] A1 subunit R7K substitution results in a wider NAD^+ binding pocket and imparts flexibility to the active site loop (A chain residues 47-56).

Table S2. DNA oligonucleotide sequences.

DNA oligo name	DNA sequence (changed bases in bold)
Mut1	CCGTTTTCACTATGGGCAAATATTATAC
Mut2	GGGCGAAGAAGTTGTCCA
Mut3	**TATAT**ATAATAGAATTAAGGATGAATTATGATTTAAG
Mut4	**TCCACT**TCAGATTGATAGCCTGAAAATATTTG
Seq1	CAA GAG ATT ACG CGC AGA CC
Seq2	CTT GGA GAG AAG AAC CCT GG
Seq3	TGC CGC GAC TCT CTA TAA TTT C
Seq4	GAT CTT GGA GCA TTC CCA CA
Seq5	TTA TAG CCA CTG CAC CCA ACA TG

References

1. Mekalanos, J. J., Collier, R. J. & Romig, W. R. Enzymic activity of cholera toxin. II. Relationships to proteolytic processing, disulfide bond reduction, and subunit composition. *J Biol Chem* **254**, 5855-61 (1979).
2. Taylor, M., Banerjee, T., Ray, S., Tatulian, S. A. & Teter, K. Protein disulfide isomerase displaces the cholera toxin A1 subunit from the holotoxin without unfolding the A1 subunit. *J Biol Chem* **286**, 22090-100 (2011).
3. Tsai, B., Rodighiero, C., Lencer, W. I. & Rapoport, T. A. Protein disulfide isomerase acts as a redox-dependent chaperone to unfold cholera toxin. *Cell* **104**, 937-48 (2001).
4. Rodighiero, C. et al. Structural basis for the differential toxicity of cholera toxin and *Escherichia coli* heat-labile enterotoxin. Construction of hybrid toxins identifies the A2-domain as the determinant of differential toxicity. *J Biol Chem* **274**, 3962-9 (1999).
5. O'Neal, C. J., Amaya, E. I., Jobling, M. G., Holmes, R. K. & Hol, W. G. J. Crystal structures of an intrinsically active cholera toxin mutant yield insight into the toxin activation mechanism. *Biochemistry* **43**, 3772-82 (2004).
6. Zhang, R. G. et al. The three-dimensional crystal structure of cholera toxin. *J Mol Biol* **251**, 563-73 (1995).
7. Sixma, T. K. et al. Refined structure of *Escherichia coli* heat-labile enterotoxin, a close relative of cholera toxin. *J Mol Biol* **230**, 890-918 (1993).
8. Merritt, E. A., Sixma, T. K., Kalk, K. H., van Zanten, B. A. & Hol, W. G. J. Galactose-binding site in *Escherichia coli* heat-labile enterotoxin (LT) and cholera toxin (CT). *Mol Microbiol* **13**, 745-53 (1994).

9. Sixma, T. K. et al. Lactose binding to heat-labile enterotoxin revealed by X-ray crystallography. *Nature* **355**, 561-564 (1992).
10. Van Den Akker, F., Steensma, E. & Hol, W. G. J. Tumor marker disaccharide D-Gal-β1,3-GalNAc complexed to heat-labile enterotoxin from *Escherichia coli*. *Protein Sci* **5**, 1184-1188 (1996).
11. van den Akker, F., Pizza, M., Rappuoli, R. & Hol, W. G. J. Crystal structure of a non-toxic mutant of heat-labile enterotoxin, which is a potent mucosal adjuvant. *Protein Sci* **6**, 2650–2654 (1997).
12. van den Akker, F. et al. The Arg7Lys mutant of heat-labile enterotoxin exhibits great flexibility of active site loop 47-56 of the A subunit. *Biochemistry* **34**, 10996-11004 (1995).
13. van den Akker, F. et al. Crystal structure of a new heat-labile enterotoxin, LT-IIb. *Structure* **4**, 665-678 (1996).
